# The Impact of Chronic Unpredictable Mild Stress-Induced Depression on Spatial, Recognition and Reference Memory Tasks in Mice: Behavioral and Histological Study

**DOI:** 10.3390/bs12060166

**Published:** 2022-05-29

**Authors:** Ghofran Khalid Alqurashi, Emad A. Hindi, Mohamed A. Zayed, Gamal S. Abd El-Aziz, Hani A. Alturkistani, Rabee F. Ibrahim, Mona Ali Al-thepyani, Refal Bakhlgi, Noor A. Alzahrani, Ghulam Md Ashraf, Badrah S. Alghamdi

**Affiliations:** 1 Neuroscience Unit, Department of Physiology, Faculty of Medicine, King Abdulaziz University, Jeddah 21589, Saudi Arabia; ghofrankhalid@hotmail.com; 2 Pre-Clinical Research Unit, King Fahd Medical Research Center, King Abdulaziz University, Jeddah 21589, Saudi Arabia; mahalthepyani@kau.edu.sa (M.A.A.-t.); rafalbakhlgi@hotmail.com (R.B.); nsalahalzahrani@stu.kau.edu.sa (N.A.A.); gashraf@kau.edu.sa (G.M.A.); 3 Department of Anatomy, Faculty of Medicine, King Abdulaziz University, Jeddah 21589, Saudi Arabia; eahindi@kau.edu.sa (E.A.H.); dr_gamal_said@yahoo.com (G.S.A.E.-A.); hturkustani@kau.edu.sa (H.A.A.); aalabrahem2@kau.edu.sa (R.F.I.); 4 Physiology Department, Faculty of Medicine, King Abdulaziz University, Rabigh 25726, Saudi Arabia; mazayed@kau.edu.sa; 5 Physiology Department, Faculty of Medicine, Menoufia University, Menoufia 32511, Egypt; 6 Department of Chemistry, College of Sciences & Arts, King Abdulaziz University, Rabigh 21911, Saudi Arabia; 7 Department of Biochemistry, Faculty of Sciences, King Abdulaziz University, Jeddah 21589, Saudi Arabia; 8 Department of Medical Laboratory Sciences, Faculty of Applied Medical Sciences, King Abdulaziz University, Jeddah 21589, Saudi Arabia

**Keywords:** depression, anxiety, stress, spatial memory, recognition memory

## Abstract

Depression-induced cognitive impairment has recently been given more attention in research. However, the relationship between depression and different types of memory is still not clear. Chronic unpredictable mild stress (CUMS) is a commonly used animal model of depression in which animals are exposed to chronic unpredictable environmental and psychological stressors, which mimics daily human life stressors. This study investigated the impact of different durations of CUMS on various types of memory (short- and long-term spatial memory and recognition memory) and investigated CUMS’ impact on the ultrastructural level by histological assessment of the hippocampus and prefrontal cortex. Twenty male C57BL/J6 mice (6 weeks old, 21.8 ± 2 g) were randomly divided into two groups (*n* = 10): control and CUMS (8 weeks). A series of behavioral tasks were conducted twice at weeks 5–6 (early CUMS) and weeks 7–8 (late CUMS). A tail-suspension test (TST), forced swimming test (FST), elevated zero maze (EZM), elevated plus maze (EPM), open field test (OFT), and sucrose-preference test (SPT) were used to assess anxiety and depressive symptoms. The cognitive function was assessed by the novel object recognition test (NORT; for recognition memory), Y-maze (for short-term spatial memory), and Morris water maze (MWM: for long-term spatial memory) with a probe test (for reference memory). Our data showed that 8 weeks of CUMS increased the anxiety level, reported by a significant increase in anxiety index in both EPM and EZM and a significant decrease in central preference in OFT, and depression was reported by a significant increase in immobility in the TST and FST and sucrose preference in the SPT. Investigating the impact of CUMS on various types of memory, we found that reference memory is the first memory to be affected in early CUMS. In late CUMS, all types of memory were impaired, and this was consistent with the abnormal histological features of the memory-related areas in the brain (hippocampus and prefrontal cortex).

## 1. Introduction

Depression is a common mental disorder that represents an important health problem worldwide. It is a known cause of global disease burden and disability [1]. According to the Diagnostic and Statistical Manual of Mental Disorders (DSM-5), the diagnosis of a major depression disorder requires the expression of five or more symptoms for at least two weeks. One of the symptoms should be either a depressed mood or anhedonia (loss of interest). The other secondary symptoms are weight or appetite changes, sleep abnormalities (hypo or hypersomnia), psychomotor impairment, fatigue or a lack of energy, a lack of concentration, feelings of excessive guilt, and suicidal ideation or attempts [2].

The prevalence of depression is about 6% of the population worldwide [3] with an approximately 18% increase in the past decade. This prevalence during a lifetime reaches about 15% [4]. This means that about one in five people experience at least one episode in their lifetime [5]. The prevalence is twice as high in women than in men and, regarding age, it rises in the second and third decades of life, with another increase in the sixth decade [6].

It is established that depression is associated with multiple disorders such as stroke [7], diabetes [8], cardiac attacks [9], and cancer [10]. Even if depression is a consequence of chronic disorders, comorbid depression is associated with the poor progression of diseases [11]. In addition, depressed patients have a higher risk of suicide [12]. Based on these findings, it is no surprise that depression is associated with a significantly higher risk of early death [13].

Depression is not only considered a health issue but also a social and economic problem. Several studies have declared that depression in students is associated with educational problems [14,15]. It is clear that the personal incomes of depressed people are significantly lower than those of people without depression [16].

Chronic exposure to multiple intermittent stressors can initiate cumulative physiological stress responses, called the “allostatic load” [17]. The allostatic load is associated with many systemic and psychological diseases [18]. Chronic stress is a psychosomatic process and a crucial risk factor for many diseases such as depression [19], cardiovascular diseases [20], and cancer [21].

The Chronic Unpredictable Mild Stress (CUMS) model is commonly used to study depression in rodents, which was first described by Willner et al. [22]. Rodents in this model are chronically exposed to constant environmentally and psychologically unpredictable mild stressors, resulting in the initiation of behavioral changes and potentially inducing depression [23,24]. This model provides a realistic depression model as it mimics daily stressors in human life and induces anhedonia, which is the core symptom of depressive disorder as mentioned in the Diagnostic and Statistical Manual of Mental Disorders IV (DSM-IV) [25,26].

Depression impairs cognitive functions during depressive and even during remission episodes [27]. Cognitive impairment in the DSM-5 classification is one of the diagnostic criteria for depression [28]. About 94% of patients expressed cognitive deficits during an episode of major depressive disorder (MDD), and 45% of them had persisting impairment during remission [29]. Another study showed impaired neural discharges in working memory in people at risk of depression even before the occurrence of depressive episodes [30]. Cortical atrophy is suggested to be the cause of cognitive impairment with depression [31], which deteriorates if not treated [32], and is expressed even in remission periods [33]. Cognitive dysfunctions lead to obvious impaired social interaction and disability. These effects create poor workplace productivity and high absenteeism and reduce the ability to achieve daily tasks.

Still, there is no clear underlying mechanism for the relationship between depression and cognitive impairment [34,35,36,37]. This effect of depression on cognitive dysfunction has a huge negative impact on the quality of life and productivity. However, no previous studies have compared and evaluated the condition of various memory types (recognition, short- and long-term spatial memory, and reference memory) with the progression of CUMS or investigated the brain pathology of responsible areas in the brain (hippocampus and prefrontal cortex). Therefore, the aim of this work is to evaluate and compare the impact of CUMS progression (early and late CUMS) on extensive behavioral analysis and various memory tasks (short- and long-term spatial, recognition, and reference memory) and investigate the effect on a structural level by histological assessment of memory-related brain areas.

## 2. Materials and Methods

### 2.1. Animal Model

Twenty male C57BL/J6 mice (6 weeks old, 21.8 ± 2 g) [38] were purchased from King Fahd Medical Research Center, King Abdulaziz University, Jeddah. The mice were kept in a temperature-controlled environment on a 12 h light/dark cycle, with free access to water and food. This work was approved by the Biomedical Ethics Committee of King Abdulaziz University (Approval No. 03-22) and followed the rules and regulations of the ACUC (animal care and use committee) in the animal house at the King Fahd Medical Research Center.

### 2.2. CUMS Procedures

Ten mice in the CUMS group were subjected to daily psychological and environmental stressors for 8 weeks as previously discussed with some modifications [39,40]. Two stressors were applied daily and chosen randomly with no repetition of the same stressor for three consecutive days to avoid any adaptation. All CUMS mice were subjected to the same type, onset, and duration of daily stressors. The time of stressor application was different every day and the time between two stressors was between 3 and 8 h. The stressors used are shown in Table 1.

### 2.3. Experimental Design

**Figure 1:**
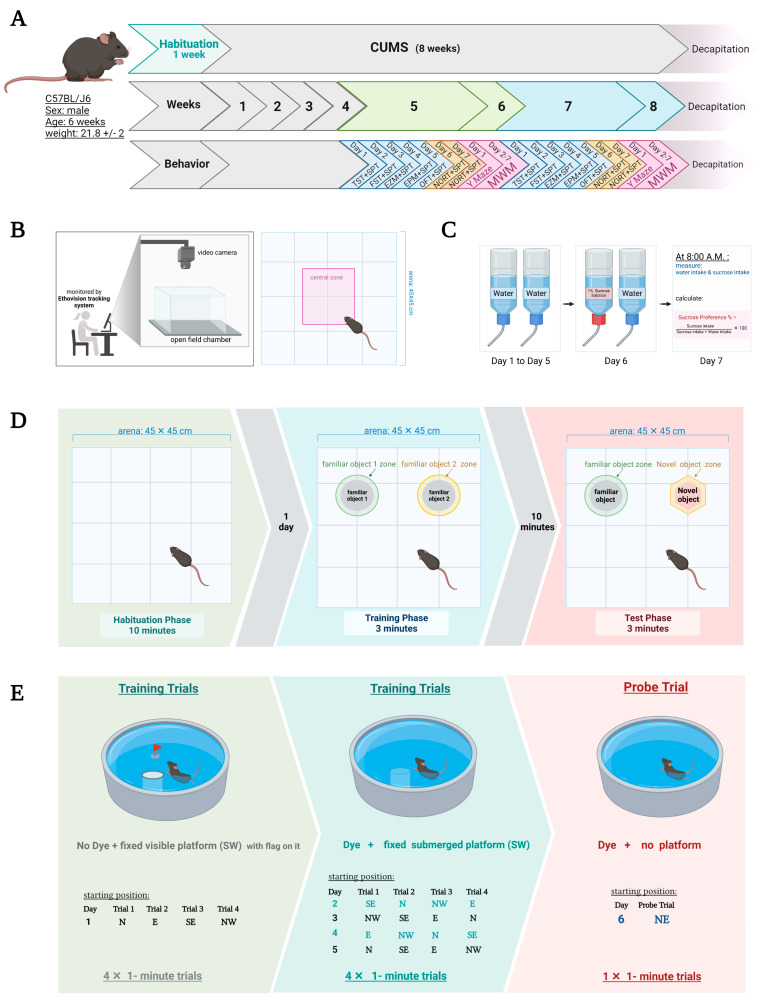
(**A**) Timeline of the experiment with an overview of behavioral tasks. The same behavioral tests were repeated in weeks 5–6 (early CUMS) and 7–8 (late CUMS). (**B**) Schematic representation of OFT under EthoVision tracking system. (**C**) SPT protocol. (**D**) NORT protocol includes 3 stages: habituation, familiarization, and test stages. (**E**) MWM protocol. CUMS: chronic unpredictable mild stress, EPM: elevated plus maze, EZM: elevated zero maze, TST: tail suspension test, FST: forced swimming test, OFT: open field test, SPT: sucrose-preference test, NORT: novel-object-recognition test, MWM: Morris water maze, N: north, E: east, SE: southeast, NW: northwest, SW: southwest, NE: northeast. Created with BioRender.com (accessed on 1 November 2021).

The mice were kept in the experimental area for 1 week of habituation. The mice were randomly divided into 2 groups (10 in each): control and CUMS. A series of behavioral tests were conducted twice in week 5 and repeated in week 7 (Figure 1A). In week 5, the following tasks were conducted: TST (tail-suspension test), FST (forced swimming test), EZM (elevated zero maze), EPM (elevated plus maze), OFT (open field test), SPT (sucrose-preference test), NORT (novel-object-recognition test) and repeated in week 7. In week 6, the Y-maze and MWM (Morris water maze) were conducted and repeated in week 8. At the end of week 8, the mice were euthanized and their brains emerged in 10% formalin for further histological analysis.

### 2.4. Weight

The weight was recorded twice weekly, and the average of each week was calculated. The percentage of weight gain was calculated as: weight gain (%) = (new weight [W1] − initial weight [W0]/initial weight [W0]) × 100 [41,42].

### 2.5. Locomotor Behavioral Tests

#### OFT

The OFT was conducted to evaluate the locomotor activity (velocity, TDM) and immobility. During the test, a mouse was placed separately in an empty arena (square test box, 45 × 45 cm) and left to move freely for 3 min. The EthoVision XT8A system (Noldus Information Technology, Wageningen, the Netherlands) was used to calculate the velocity, TDM, and immobility.

### 2.6. Anxiety Behavioral Tests

#### 2.6.1. Central Preference %

An EthoVision system was also used in the OFT to calculate the duration spent in the central zone (Figure 1B). The percentage of central-zone preference was calculated as follows: central preference % = (time in central zone/total experiment time) × 100 [41].

#### 2.6.2. EZM

The EZM was used to measure anxiety-like behavior, which is associated with depression models. The apparatus comprised an elevated circular platform with two opposite, enclosed quadrants and two open quadrants [43]. The anxiety index was measured by the following equation: anxiety index = 1 − ([open arm time/5 min] + [open arm entry/total entry])/2.

#### 2.6.3. EPM

The EPM was used to measure anxiety-like behavior, which is associated with depression models. The apparatus consisted of 4 elevated arms in plus shape in which 2 of them were open and the other 2 closed. The mouse was placed in the center and its behavior recorded for 5 min. The anxiety index was measured by the following equation: anxiety index = 1 − ([open arm time/5 min] + [open arm entry/total entry])/2 [41].

### 2.7. Depression Behavioral Tests

#### 2.7.1. TST

The TST was used to evaluate depression-like behaviors in mice. Each mouse was suspended by it’s tail with adhesive tape, 50 cm above the bench. Mice were suspended for 6 min, and the immobility time was recorded in the last 4 min of the experiment [44]. Immobility is a depression-like behavior and was considered to be when the mouse was passively suspended with no movement at all [44].

#### 2.7.2. FST

The FST was used to evaluate depression-like behaviors in the mice. Each mouse was placed in a separate glass cylinder (20 cm × 40 cm) filled with 30 cm-high warm water (25 ± 1 ℃). The total experiment time was 6 min, and the immobility time was recorded in the last 4 min [44]. Immobility was considered when the mouse passively floated in an upright position to keep his nose above the water with no struggling or movement [44].

#### 2.7.3. SPT

The SPT was used to measure anhedonia, which is a characteristic sign of depression. We calculated the preference for a 1% sucrose solution by using a two-bottle-choice test. Mice were kept in cages with two bottles of water for 5 days at the adaptation stage. Then, during the test phase on day 6, mice were housed individually in cages with two bottles of 1% sucrose and water for 12 h [44] (Figure 1C). The location of bottles was switched after 6 h to avoid any side preference. The consumption of sucrose was measured by the following equation: sucrose preference % = (the milliliters consumed from the sucrose bottle/the milliliters consumed from the water bottle + the milliliters consumed from the sucrose bottle) × 100 [41,44].

### 2.8. Memory Tests

#### 2.8.1. NORT

The NORT is a commonly used test to evaluate recognition memory. It consisted of 3 stages and was conducted over 2 days. Day 1 was the habituation stage in which each mouse was placed in an open arena for 15 min. Day 2 was the familiarization stage in which the mouse was subjected to 2 identical objects (familiar object 1 (F1) and familiar object 2 (F2)) and left for 3 min to be given the chance to explore these objects [45]. After 10 min, one of the familiar objects was replaced by a novel object the mouse was allowed to explore for another 3 min (Figure 1D). The EthoVision system calculated the duration spent in each zone and the frequency of visiting the nose point (sniffing) in each zone [45]. The discrimination index (DI) was calculated according to the following equation: DI = (TN − TF)/(TN + TF), in which TN is the time spent on the novel object and TF is the time spent on the familiar object [42].

#### 2.8.2. Y-Maze

Spontaneous alternation is a common test used to evaluate short-term spatial memory using a Y-maze. The apparatus consisted of 3 symmetrical 60 cm-long, 10 cm-wide arms that were each ascribed a letter (A, B, and C). The mouse was placed in one arm and left to freely move for 5 min. The sequence of arm entries was recorded for the whole experiment. The spontaneous alternation score was given when there was a sequential entry into the three different arms (i.e., ABC, CBA, or BAC). The spontaneous alternation percentage (SA%) was calculated by SA% = number of alternations/(total arm entries − 2) × 100 [41].

#### 2.8.3. MWM

The MWM is a circular open pool filled halfway with water with a featureless interior surface. The mice were allowed to swim and search for the target (submerged hidden platform), which was in a fixed position (Figure 1E). Several training trials were performed to help the mice learn how to escape and locate the site of the platform depending on distal cues for navigation (see Figure 1E). The platform was camouflaged by adding opacifying non-toxic dye in the water to reduce any visual clues seen by the animal underwater when swimming [41]. In each trial, the mice were given the chance to search for an underwater platform for a maximum of 60 s [46]. The time needed for the mice to reach the platform was calculated as the escape latency [41]. The probe trial was performed on the sixth day to assess the reference memory [47]. The exact protocol and training trials are shown in Figure 1E.

### 2.9. Tissue Harvesting and Processing

Brains were harvested, mid-sagittally dissected into 2 hemispheres and then fixed in 10% formalin for 48 h. The tissues were processed overnight by using the Spin Tissue Processor STP120 (Especialidades Médicas Myr, S.L., Tarragona, Spain). The brain specimens were embedded in paraffin wax, and brain sections were cut (4 µ in thickness) using a LEICA RM 2255 (Leica Microsystems, Germany) and mounted onto glass slides for staining. The slides were stained with eosin and hematoxylin to elucidate the pathohistological changes in CUMS models using Myr AutoStainer (Especialidades Médicas Myr, S.L., Tarragona, Spain). Histological images were snapped from the frontal cortex and hippocampus regions by using an Olympus BX53 microscope with an Olympus DP73 camera at different powers, and images were processed by using Olympus CellSens Entry software (Olympus Corporation, Tokyo, Japan).

### 2.10. Statistical Analysis

The data were analyzed using GraphPad Prism 8 (GraphPad Inc., La Jolla, CA, USA). Data are shown as the mean ± standard error of the mean (mean ± SEM). A two-way analysis of variance (ANOVA) was conducted to analyze the results. Šídák’s multiple-comparisons test was used as a post hoc test for significant ANOVA results, to compute confidence intervals for every comparison. Differences with *p* < 0.05 were considered to be statistically significant.

## 3. Results

### 3.1. Effect of CUMS on Body Weight

**Figure 2:**
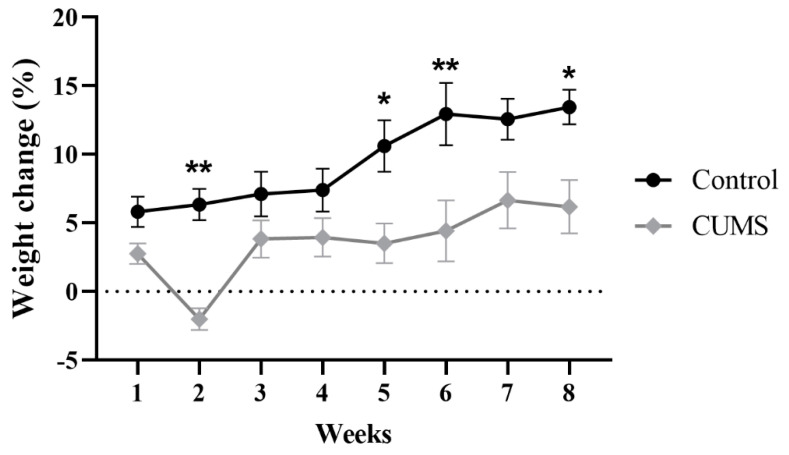
The effect of CUMS on the weekly percentage of weight change. Data are presented as mean ± standard error of the mean (SEM). Two-way repeated-measures ANOVA was used, followed by Šídák’s multiple-comparisons test. (*) indicates a significant difference between the CUMS and the control group at *p* > 0.05 and ** *p* < 0.01.

Two-way repeated-measures ANOVA showed a significant effect of weeks × groups (F(7, 114) = 2.662, *p* = 0.0137), weeks (F(7, 114) = 17.36, *p* < 0.0001), and groups (F (1, 17) = 9.895, *p* = 0.0059) (Figure 2). Further analysis using Šídák’s multiple-comparisons post hoc test revealed that CUMS significantly reduced the percentage of weight gain compared with the control group at week 2 (*p* = 0.0014), week 5 (*p* = 0.0108), week 6 (*p* = 0.0024), and week 8 (*p* = 0.0411) (Figure 2).

### 3.2. Effect of Early and Late CUMS on Locomotor Activity

**Figure 3:**
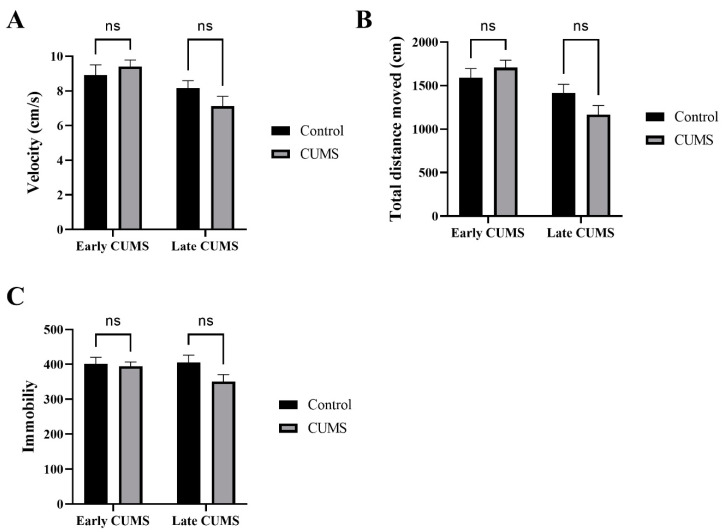
The effect of early and late CUMS on locomotor activity in the OFT. (**A**) Velocity (cm/s); (**B**) total distance moved (cm); (**C**) immobility frequency. Two-way ANOVA was used, followed by Šídák’s multiple-comparisons test. OFT: open field test; ns: not significant.

The effects of CUMS on locomotor activity were measured by the OFT, which calculated the velocity, TDM, and immobility. Two-way ANOVA with Šídák’s post hoc test revealed that CUMS did not affect the velocity, TDM, or immobility in both early (*p* = 0.7489, *p* = 0.6608, *p* = 0.9535, respectively) and late (*p* = 0.2846, *p* = 0.1623, *p* = 0.0906) CUMS compared with the control group (Figure 3A–C).

### 3.3. Effect of Early and Late CUMS on Anxiety Behavior

**Figure 4:**
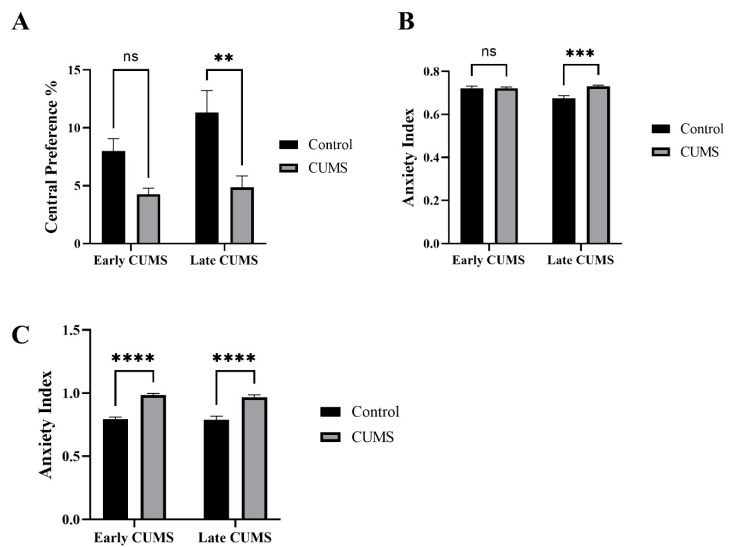
The effect of early and late CUMS on anxiety in (**A**) central preference percentage, (**B**) EZM, and (**C**) EPM. Two-way ANOVA was used, followed by Šídák’s multiple-comparisons test. (**) indicates a significant difference between the CUMS groups and the control group at *p* < 0.01, *** *p* < 0.001, and **** *p* < 0.0001. ns: not significant.

The effects of CUMS on anxiety were measured by the central preference in the OFT and anxiety index in both EPM and EZM (Figure 4A–C). In the OFT, CUMS significantly reduced the central preference in late (*p* = 0.0023) but not early (*p* = 0.1082) CUMS compared to the control group (Figure 4A). In EZM, the anxiety index was significantly increased in the CUMS group compared to the control in late (*p* = 0.0004) but not early CUMS (*p* = 0.9990) (Figure 4B). In the EPZ, CUMS significantly increased the anxiety index in both early (*p* <0.0001) and late (<0.0001) CUMS compared to the control (Figure 4C).

### 3.4. Effect of Early and Late CUMS on Depression Behavior

**Figure 5:**
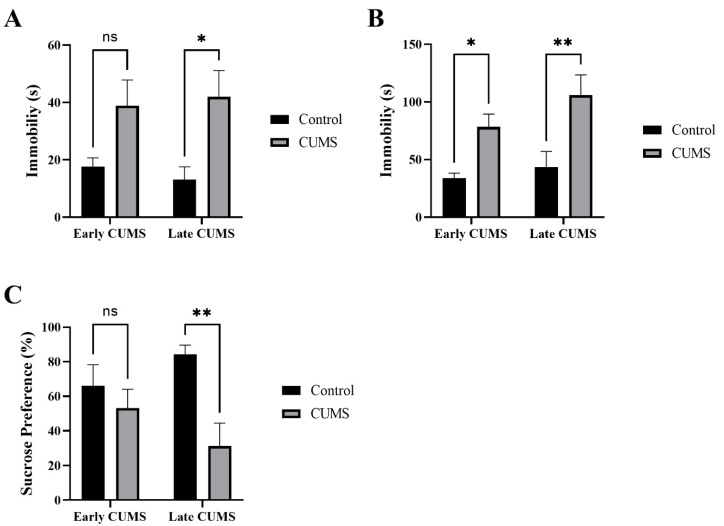
The effect of early and late CUMS on depression tests in the (**A**) TST, (**B**) FST, and (**C**) SPT. Two-way ANOVA was used, followed by Šídák’s multiple-comparisons test. (*) indicates a significant difference between the CUMS groups and the control group at *p* > 0.05, ** *p* < 0.01. ns: not significant.

The effects of CUMS on depression-like behavior were measured by the immobility time in the TST, FST, and SPT (Figure 5A–C). In the TST, CUMS significantly increased the immobility time in late (*p* = 0.0192) but not early (*p* = 0.1000) CUMS compared with the control (Figure 5A). In the FST, CUMS significantly increased the immobility time in both early (*p* = 0.0310) and late (*p* = 0.0022) CUMS compared with the control (Figure 5B).

In the SPT, the CUMS group consumed significantly less sucrose than the control group in late (*p* = 0.0041) but not early (*p* = 0.6328) CUMS (Figure 5C).

### 3.5. Effect of Early and Late CUMS on Memory

#### 3.5.1. Effect of Early and Late CUMS on Spatial Memory

The effects of CUMS on spatial memory were evaluated by the Y-maze for short-term spatial memory and the MWM for long-term spatial memory.

**Figure 6:**
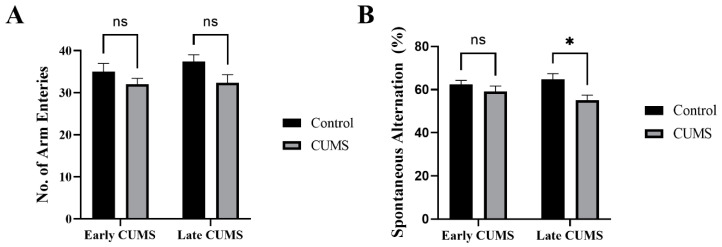
The effect of early and late CUMS on short-term spatial memory in the Y-maze task: (**A**) number of arm entries and (**B**) spontaneous alternation. Two-way ANOVA was used, followed by Šídák’s multiple-comparisons test. (*) indicates a significant difference between the treated CUMS and the control group at *p* > 0.05. ns: not significant.

In the Y-maze, there was no significant difference between the number of arm entries in the CUMS group during early (*p* = 0.4158) and late (*p* = 0.0961) CUMS compared with the control group (Figure 6A). CUMS significantly reduced the spontaneous alternation percentage in late (*p* = 0.0131) but not early (*p* = 0.5581) CUMS compared with control (Figure 6B).

**Figure 7:**
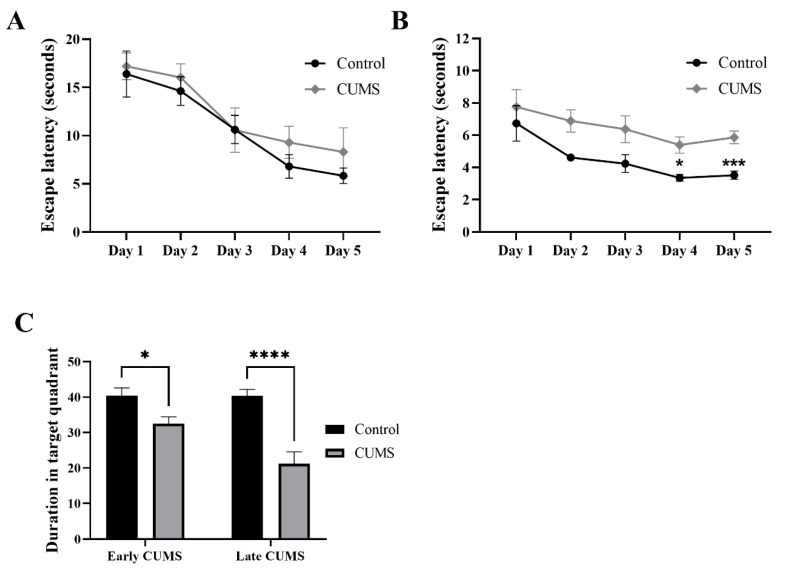
The effect of CUMS on long-term spatial memory in the MWM task. The effect of early (**A**) and late (**B**) CUMS on escape latency time and (**C**) probe test. Two-way ANOVA was used, followed by Šídák’s multiple-comparisons test. (*) indicates a significant difference between the CUMS and the control group at *p* > 0.05, *** *p* < 0.001, and **** *p* < 0.0001.

In the MWM of early CUMS, two-way repeated-measures ANOVA showed no significant effect of days × groups (F (4, 68) = 0.2310, *p* = 0.9201) or groups (F (1, 17) = 1.083, *p* = 0.3126), but a significant effect of days (F (3.219, 54.72) = 14.53, *p* < 0.0001) (Figure 7A). Further analysis using Šídák’s multiple-comparisons post hoc test revealed that CUMS did not affect the latency time throughout the 5 days of the experiment compared to the control (Figure 7A).

On the other hand, in the MWM of late CUMS, two-way repeated-measures ANOVA showed no significant effect of days × groups (F (4, 68) = 0.3457, *p* = 0.8462), but a significant effect of groups (F (1, 17) = 20.55, *p* = 0.0003) and days (F (2.185, 37.14) = 6.009, *p* = 0.0045) (Figure 7B). Further analysis using Šídák’s multiple-comparisons post hoc test revealed that CUMS significantly increased the latency time on day 4 (*p* = 0.0181) and 5 (*p* = 0.0009) compared to the control (Figure 7B).

In the probe test, CUMS significantly reduced the duration in the target quadrant in both early (*p* = 0.0464) and late (*p* < 0.0001) CUMS compared to the control (Figure 7C).

#### 3.5.2. Effect of Early and Late CUMS on Recognition Memory

**Figure 8:**
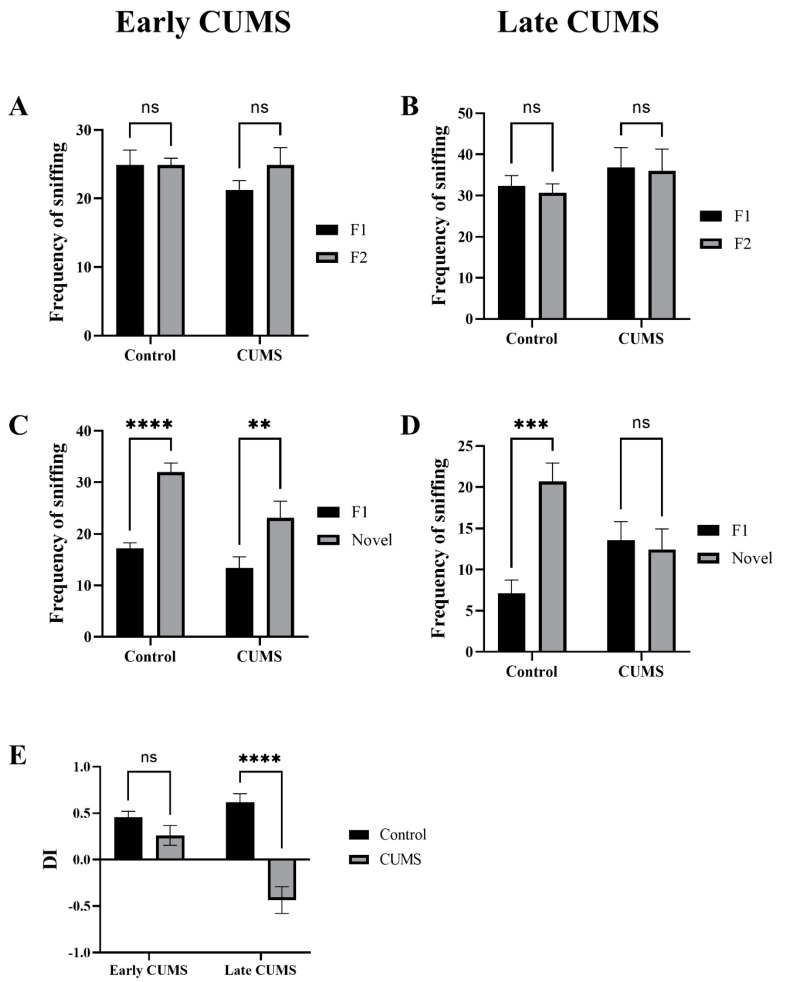
The effect of CUMS on recognition memory in the NORT task. The frequency of sniffing familiar objects (F1 and F2) in the familiarization stage in early (**A**) and late (**B**) CUMS. The frequency of sniffing objects (F1 and novel) in the test stage in early (**C**) and late (**D**) CUMS. The discrimination index (DI) is shown in (**E**). Two-way ANOVA was used, followed by Šídák’s multiple-comparisons test. (**) indicates a significant difference between the CUMS and the control group at *p* < 0.01, *** *p* < 0.001, and **** *p* < 0.0001. ns: not significant.

In early CUMS, there was no significant difference in the frequency of sniffing familiar objects (F1 and F2) during the familiarization phase between the control (*p* > 0.9999) and CUMS groups (*p* = 0.3515) (Figure 8A). Similarly, in late CUMS, there was no significant difference in the frequency of sniffing familiar objects (F1 and F2) during the familiarization phase between the control (*p* = 0.9429) and CUMS groups (*p* = 0.9848) (Figure 8B).

In early CUMS, there was a significant increase in the frequency of sniffing the novel object (novel) during the test phase in the control (*p* < 0.0001) and CUMS groups (*p* = 0.0065) (Figure 8C). However, in late CUMS, there was a significant increase in the frequency of sniffing the novel object (novel) during the test phase only in the control (*p* = 0.9429) and non-CUMS groups (*p* = 0.9848) (Figure 8D).

The CUMS group showed significantly less DI than the control group in late (*p* <0.0001) but not early (*p* = 0.3099) CUMS (Figure 8E).

### 3.6. Effect of CUMS on the Brain Histological Feature

**Figure 9:**
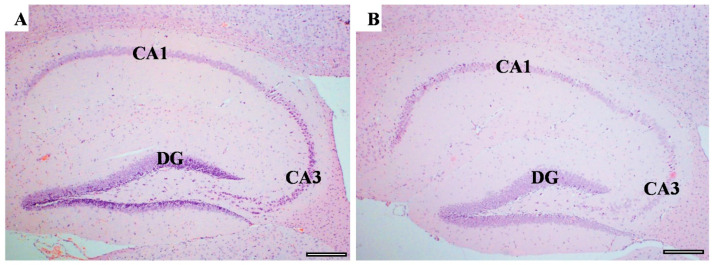
The hippocampus structure. H&E staining of hippocampus area sagittal section in both controls (**A**) and CUMS (**B**). The hippocampus areas CA1, CA3, and the dentate gyrus (DG) are the main areas that are involved in memory function. There is no interstitial bleeding or infiltration of inflammatory cells in CUMS group. Scale bar = 200 µm.

**Figure 10:**
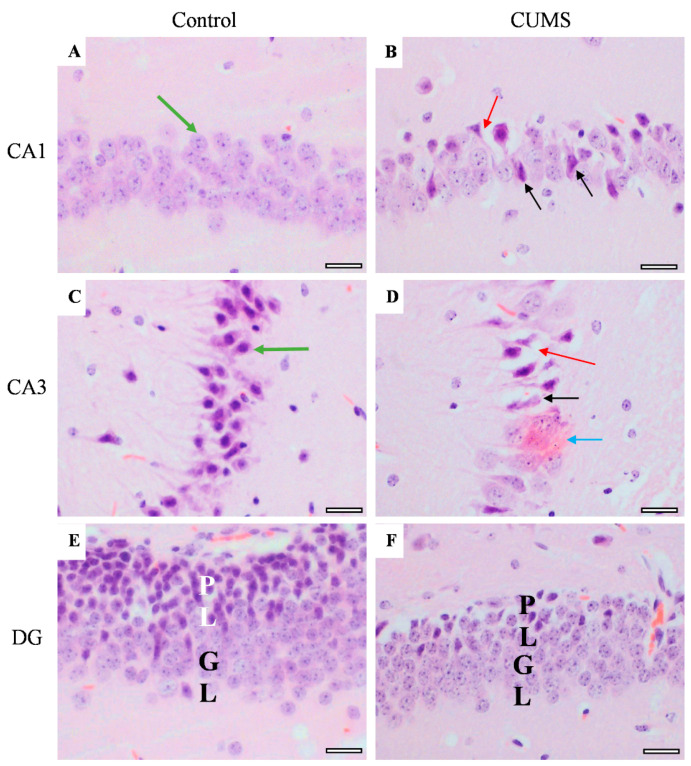
The effect of CUMS on the hippocampal regions. Representative images of hippocampus areas (CA1 and CA3) and dentate gyrus (DG) from control (**A**,**C**,**E**) and CUMS (**B**,**D**,**F**). The controls had normal neurons with vesicular pale nuclei in CA1 (green arrow) (**A**) while the control CA3 showed normal large basophilic neurons with dark vesicular nuclei and clear axons (green arrow) (**C**). However, both CA1 and CA3 in CUMS (**B**,**D**, respectively) had degenerated neurons (black arrow) and loss of tissue surrounding the degenerated neurons (red arrow). A dark eosinophilic plaque (blue arrow) was observed in CUMS CA3 (**D**). The dentate gyrus (DG) polymorphic layer (PL) is thinner in CUMS (**E**) compared with controls (**F**). There was no significant change in the DG granular layer (GL) in both groups. Scale bar = 20 µm.

**Figure 11:**
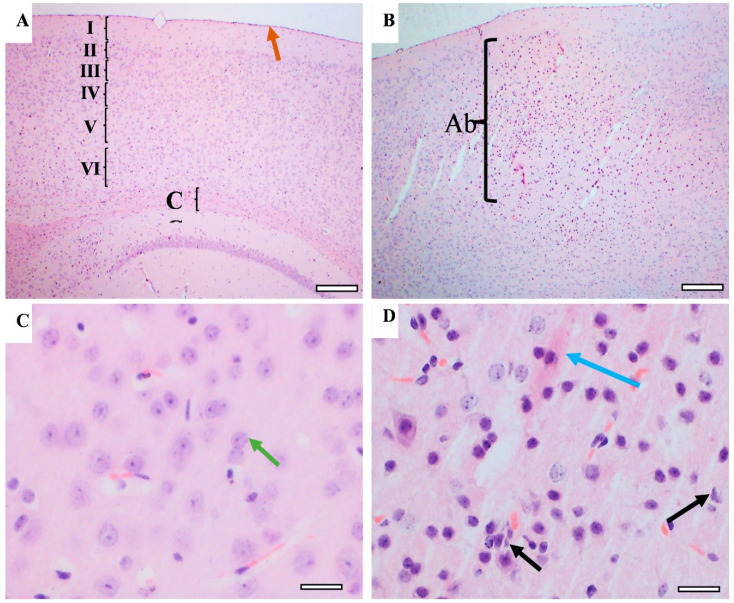
The effect of CUMS on the frontal cortex. Representative frontal cortex areas stained with H&E from controls (**A**,**C**) and CUMS (**B**,**D**). The control frontal cortex showed the pia matter (brown arrow) and 6 cortical layers (from I to VI) and part of the corpus callosum (CC) in low-power images (**A**). The cortical layers disrupted by abnormal cellular overgrowth area (Ab) in CUMS frontal cortex (**B**). The high-power image observed normal neurons with large vesicular nuclei and clear nucleolus and chromatin materials in the control frontal cortex (green arrow) (**C**). The cellular overgrowth area in CUMS cortex contains degenerated neurons (black arrow), eosinophilic extracellular plaques (blue arrow), and neurons with dark basophilic nuclei without clear nucleolus. (**D**). Scale bar A,B = 200 µm, C,D = 20 µm.

Memory-related areas (hippocampus and frontal cortex) were examined histologically under a microscope to identify the histopathological changes. In the hippocampus, the low-power images did not show significant changes in CUMS compared with the controls (Figure 9). However, a high-power examination obtained neuronal degeneration in both CA1 and CA3 associated with a loss of extracellular tissues surrounding the degenerated neurons (Figure 10). In addition, the formation of a dense eosinophilic area in CA3 was observed. The CUMS group’s dentate gyrus had fewer polymorphic cells in the dentate gyrus compared with the controls (Figure 10). The six frontal cortex layers—I (molecular), II (external granular), III (external pyramidal), IV (internal granular), V (internal pyramidal), and VI (multiform)—were easily observed in the low-power images (Figure 11). These layers were disrupted in the CUMS group by a dense area with dark basophilic cells. Upon the high-power examination, the area included many neurons with small, dark basophilic nuclei and unclear chromatin materials, and also degenerated neurons. A dense, irregular, ill-defined extracellular area was also observed in the abnormal cortical area (Figure 11).

## 4. Discussion

The effect of depression on cognitive dysfunction has a huge negative impact on the quality of life and productivity with no clear underlying mechanism [34,35,36,37]. However, no previous studies have compared the impact of CUMS on extensive behavioral outcomes and evaluated the condition of various memory types (recognition, short- and long-term spatial memory, and reference memory) with the progression of CUMS. Therefore, the aim of this work is to evaluate and compare the impact of CUMS progression (early and late CUMS) on extensive behavioral analysis (locomotor, anxiety, and depression) and various memory tasks (short- and long-term spatial, recognition, and reference memory) and investigate the impact on a structural level by histological assessment of memory-related brain areas (hippocampus and prefrontal cortex).

Our results revealed decreased weight gain in CUMS-exposed mice over weeks of the experiment when compared with a control group. This result is in agreement with previous studies performed on animals exposed to chronic stress [48,49]. A reduction in weight gain in the CUMS group was shown as an indicator of the stress response that led to the initiation of anhedonia [49,50,51].

In our study, there was a non-significant change in locomotor behavior (velocity, TDM, immobility) measured by OFT in either early or late CUMS exposure. This result is contrary to study results that exhibited psychomotor inhibition, as noted in MDD patients and experimental CUMS rodents with or without hedonic impairment [52,53,54]. Nevertheless, some studies reported similar results to our study, with either a change in the open field behavior or even an increase in locomotion activity [55] that contradicted the psychomotor impairment as a characteristic of depression [56]. Different factors can affect locomotion, such as illumination; a previous study found that 25 Lux can promote hyperlocomotion, while 5 Lux showed no significant difference in the OFT results of TDM in chronically stressed mice compared with control [57,58].

Anxiety is considered a comorbid state independent of depression. Many studies declared high anxiety in chronic unpredictable stress [59,60], which was consistent with our research. Any abnormality in psychomotor behavior such as hyperlocomotion directly affects anxiety measures as present in some studies [61,62]. However, in our study, there was a reduction in central preference and an increase in anxiety index assessed by the EZM and EPZ with no difference in locomotion.

Moreover, depression was demonstrated by a significant increase in immobilization in the FST and TST. These results agreed with different studies that showed an increased immobilization time, which was considered a measure of behavioral despair [63,64,65]. This prolonged time of immobility in the TST and FST can be reversed using antidepressants [66,67]. Importantly, studies declared that anhedonia was linked to an increased immobility time [68].

In an interesting study, there was a prolonged time of immobility in the FST and TST in stressed mice following 3 weeks of CUMS exposure. However, these parameters were decreased after 5 weeks, which indicates the presence of adaptation [48].

Anhedonia is considered the essential sign for depression models, and it is assessed by a reduction in sucrose preference and consumption [69]. In the current study, we have used a CUMS mouse model to mimic human daily stressors, which is confirmed by different behavioral tasks, especially the SPT, which is considered a well-validated test for mouse models for depression [26]. In our study, there was a significantly lower sucrose preference in prolonged but not early chronic unpredictable stress exposure. This result is consistent with other studies that revealed significantly decreased sucrose preference at the seventh week or even more of CUMS [70,71], while other studies declared lower sucrose preference earlier at 3 weeks [72,73,74] and 5 weeks [75,76]. This variability of results depends on multiple factors such as rodent strain, water, and food deprivation before testing, the concentration of sucrose solution, and circadian rhythm in applying the test.

There is an association between depression and memory impairment. Our data showed that the first type of memory that could be affected by CUMS in the early stages of CUMS is the reference memory, which was tested by the probe test in the MWM. This result is coincident with previous studies that reported that 4 weeks [39] and 6 weeks [77] of CUMS significantly reduced the time in the targeted quadrant in the probe test. Moreover, our data showed that reference memory is affected before the SPT symptoms started, which is consistent with previous work [77].

In the late stages of CUMS, dramatic changes in all other memory tasks significantly appeared with the coincidence of a significant change in the SPT. Our data showed that short-term spatial memory tested by spontaneous alternation deteriorated in the late stages of CUMS. However, previous work has reported that 4 weeks of CUMS applied to adult male Kunming mice were enough to alter the spontaneous alternation percentage [39]. However, they also reported that 4 weeks of CUMS was sufficient to significantly change the SPT [39]. These changes could be due to mouse species differences.

Our data showed that long-term spatial memory tested by escape latency in the MWM deteriorated only in the late stages of CUMS with the appearance of the SPT symptoms. The effect of CUMS on the MWM was variable in previous works, in which some found that 4 weeks was enough to induce memory impairment in adult male Sprague Dawley rats [78] and adult male Kunming mice [39], with a significant change in the SPT as well. Others have found that 5 weeks of CUMS induced MWM task impairment in adult male Wistar rats with no data regarding the SPT [79]. Moreover, a study found that 6 weeks of CUMS was essential to cause MWM task impairment in adult male Sprague Dawley rats with no change in the SPT [77]. These differences could be due to the rodent type, species, and CUMS protocol difference.

Not enough studies have investigated the effect of CUMS on recognition memory [80,81,82]. The NORT is a well-known test that relies on rodents’ natural exploring novelty and no need for negative or positive reinforcements (e.g., food or electric shocks) or any long training schedule [83]. This makes the NORT comparable to memory tests currently used in humans and increases the ecological validity of the test. Moreover, the NORT does not involve reference memory components (e.g., explicit rule learning); thus, it can be considered a “pure” recognition memory test and a valid task to assess working memory. It is considered a suitable behavioral assay for depression models as it has lower stress compared with other memory tests [40,84,85,86,87,88]. Our data have shown that late stages of CUMS, but not early, induced recognition memory impairment measured by the DI of the NORT. The frequency of sniffing was calculated to ensure that all mice had the chance to explore both objects (F1 and F2) equally in the familiarization stage. Moreover, our data showed that mice in the CUMS group had a similar sniffing frequency for both objects in the test phase (F1 and novel objects), which rules out any possibility of novelty-induced anxiety [89,90]. The DI was significantly different between CUMS and control only in the late CUMS stage. This is consistent with previous studies that found that 5 weeks of CUMS significantly decreased the DI in male Wistar rats [79]. Another study has found that 35 days of CUMS significantly changed the DI of the NORT in male Kunming mice [81]. The variation of outcome is wide because of different species, CUMS protocols, and the validity of depression models. In our study, we rely on the SPT to make sure we have reached the important hallmark symptom of depression.

Chronic stress creates structural changes in depressed brains such as a decrease in the hippocampal volume [91], which is associated with CUMS-induced anhedonia [92]. In addition, exposure to CUMS causes a lowered expression of brain-derived neurotrophic factor (BDNF) [93] and other neurotrophins [94]. This reduction in trophic action causes the shrinkage of neuronal dendrites of the hippocampus [95] and the loss of granular cells [96]. Additionally, the hippocampus, unlike most other brain areas, expresses neurogenesis that is continuous in adult life, and this action is inhibited by prolonged exposure to stressors including CUMS [97].

## 5. Conclusions

In conclusion, our data shed light on the serious impact of depression on cognitive dysfunction, which could mediate the huge negative impact on the quality of life and productivity. Our data reported that reference memory is the first type of memory to be affected by CUMS at the early stages (early CUMS). This is followed by impairment of other types of memory including spatial (short- and long-term) and recognition memory in the late stages of CUMS. This is associated with brain pathology in which histological examination demonstrated neuronal degeneration in the hippocampus and disruption in the prefrontal cortex. In the current study, some tests were significant in early CUMS but the majority showed slight changes which did not reach a significant level. The behavioral results showed some variation, and this is acceptable, especially in behavioral tests known to have a wide variation. So, we conclude that a longer duration of CUMS can induce impairment in all tests. These data highlighted the serious complications of CUMS, which mimics human daily life stressors if no intervention was applied. More studies are needed to investigate the molecular mechanism underlying neurocognitive impairment in CUMS. Quantitative histological assessment for neurodegeneration is also required in different brain regions. We hope to stimulate future studies to suggest and investigate new treatment avenues or intervention strategies to improve the chronic stress outcomes in daily life which could improve the quality of life and productivity.

## Figures and Tables

**Table 1:** Stressors used in the Chronic Unpredictable Mild Stress (CUMS) model.

	Stressor	Duration	Description	Days of CUMS Experiment
1	Tail clamping	1 min	Tail pinch 1 cm from the distal part of the tail	1, 8, 13, 23, 27, 32, 37, 47, 51, 55
2	Restraining	4 h	Restrain in an air-permeability tube	2, 7, 18, 21, 25, 33, 40, 38, 44, 49
3	Wet cage	24 h	A cage with damp bedding with water	3, 8, 13, 22, 26, 31, 39, 46, 51, 56
4	Food or water deprivation	24 h	No water bottles or food pellets	4, 7, 14, 20, 25, 32, 42, 45, 49, 55
5	Tilted cage (30° degree)	24 h	Cages are elevated from one side and kept tilted at 30° degree.	1, 10, 16, 19, 28, 35,39, 43, 50, 56
6	Illumination	12 h	Overnight illumination (lights on overnight)	2, 9, 18, 24, 27, 34, 38, 48, 52
7	Isolation	24 h	Housing in separate cages (1 mouse/cage)	6, 11, 15, 21, 30, 36, 40, 44, 53
8	Cage shaking	10 min	Cage shaking (200 rpm)	3, 9, 15, 19, 26, 31, 37, 43, 52
9	Predator sounds	10 min	Exposure to loud predator sounds	4, 12, 16, 20, 29, 35, 42, 45, 50
10	An empty cage	24 h	Empty cage with no bedding	5, 12, 17, 23, 29, 33, 41, 47, 54
11	Exposure to an empty water bottle	1 h	Water bottle is replaced by an empty one	6, 10, 14, 24, 28, 36, 40, 48, 53
12	Swimming in cold water (4 ℃)	3 min	Forced swimming in cold water (4 ℃) in cylinder.	5, 11, 17, 22, 30, 34, 41, 46, 54

## Data Availability

Not applicable.
